# Abundance and distribution of RNA polymerase II in *Arabidopsis* interphase nuclei

**DOI:** 10.1093/jxb/erv091

**Published:** 2015-03-04

**Authors:** Veit Schubert, Klaus Weisshart

**Affiliations:** ^1^Leibniz Institute of Plant Genetics and Crop Plant Research (IPK) Gatersleben, D-06466 Stadt Seeland, Germany; ^2^Carl Zeiss Microscopy GmbH, D-07743 Jena, Germany

**Keywords:** *Arabidopsis thaliana*, endopolyploidy, photoactivated localization microscopy, RNA polymerase II, structured illumination microscopy, transcription factory.

## Abstract

RNA polymerase II (RNAPII) is responsible for the transcription of most eukaryotic protein-coding genes. Analysing the topological distribution and quantification of RNAPII can contribute to understanding its function in interphase nuclei. Previously it was shown that RNAPII molecules in plant nuclei form reticulate structures within euchromatin of differentiated *Arabidopsis thaliana* nuclei rather than being organized in distinct ‘transcription factories’ as observed in mammalian nuclei. Immunosignal intensity measurements based on specific antibody labelling in maximum intensity projections of image stacks acquired by structured illumination microscopy (SIM) suggested a relative proportional increase of RNAPII in endopolyploid plant nuclei. Here, photoactivated localization microscopy (PALM) was applied to determine the absolute number and distribution of active and inactive RNAPII molecules in differentiated *A. thaliana* nuclei. The proportional increase of RNAPII during endopolyploidization is confirmed, but it is also shown that PALM measurements are more reliable than those based on SIM in terms of quantification. The single molecule localization results show that, although RNAPII molecules are globally dispersed within plant euchromatin, they also aggregate within smaller distances as described for mammalian transcription factories.

## Highlight

Super-resolution microscopy reveals the number of RNA polymerase II molecules in plant interphase nuclei. Both active and inactive polymerase variants aggregate in a range known from mammalian transcription factories.

## Introduction

Most eukaryotic genes are transcribed by RNA polymerase II (RNAPII) (Kornberg, 1999; Sims *et al.*, 2004). Post-translational modifications of the C-terminal domain (CTD) of the largest subunit of RNAPII are important for controlling transcription, mRNA processing, chromatin remodelling, and RNA export (Hajheidari *et al.*, 2013). The quantity of active RNAPII in nuclei reflects the degree of transcription. The co-localization of transcripts and RNAPII has been proven by electron (Iborra *et al.*, 1996) and light microscopy (Grande *et al.*, 1997; Osborne *et al.*, 2004). Depending on its position on a gene and the stage of transcription, RNAPII is differentially phosphorylated. Inactive RNAPII is mainly unphosphorylated. Its activation requires the phosphorylation at Ser2 and Ser5 of the heptapeptide YSPTSPS present as tandem repeats in the RNAPII CTD (Hirose and Ohkuma, 2007; Hajheidari *et al.*, 2013). Antibodies specific for the phosphorylation state of the peptide allow the discrimination between active and inactive RNAPII (Bourdon *et al.*, 2012). To initiate transcription and for the binding onto promoters of genes, phosphorylation at Ser5 is necessary (Hengartner *et al.*, 1998; Komarnitsky *et al.*, 2000). For the elongation step of transcription, phosphorylation at Ser2 is required (Ni *et al.*, 2004). At the end of transcription, the Ser5 phosphorylation is removed, whereas RNAPIISer2ph accumulates at the 3ʹ end of the genes (Egloff and Murphy, 2008).

In mammals, RNAPII is thought to be organized in distinct so-called ‘transcription factories’ (Jackson et al., 1993, 1998; Chakalova and Fraser, 2010; Ferrai *et al.*, 2010; Rieder *et al.*, 2012; Papantonis and Cook, 2013). In human and murine cells, the size of transcription factories ranges between ~40nm and 200nm in diameter and increases with higher transcriptional activity (Iborra *et al.*, 1996; Eskiw *et al.*, 2008; Eskiw and Fraser, 2011). The number of transcription factories in a nucleus seems to be cell type and species specific.

In mouse nuclei of different cell types, ~100–1500 transcription factories were found, whereas those in mouse and human erythroblasts amounted to ~100–550 and ~1500, respectively (Osborne *et al.*, 2004; Brown *et al.*, 2008; Eskiw and Fraser, 2011).

These numbers may also vary depending on the imaging technique applied. In HeLa cells (a human tumour cell line) ~300–500 factories were identified by widefield fluorescence microscopy (Jackson *et al.*, 1993), ~2100 by combining electron and confocal microscopy (Iborra *et al.*, 1996), ~2400 by electron microscopy alone (Jackson *et al.*, 1998), and ~850–3900 by deconvolution microscopy (Fay *et al.*, 1997). Pombo *et al.* (1999) found ~10 000 RNAPIII foci by cryo-sectioning. In addition, the methodologies detecting different molecules related to transcription (e.g. mRNA, RNA polymerase, and splicing factors) could also induce the variability in the number of foci detected.

In a single transcription factory, the number of RNAPII molecules ranges from four to 30 (Iborra *et al.*, 1996; Jackson *et al.*, 1998; Martin and Pombo, 2003). These numbers convert to a minimum of ~400 (100×4) and a maximum of ~117 000 (3900×30) RNAPII molecules per nucleus, based on the estimation of 100–3900 factories per nucleus. Obviously, this high variability was not only caused by the different cell types analysed, but rather by the different imaging methods applied.

The recently developed super-resolution microscopy techniques such as photoactivated localization microscopy (PALM) and spectral position determination microscopy (SPDM) to localize single molecules at high precision beyond the diffraction limit of light allow a more reliable counting of molecules in cells (Lando *et al.*, 2012; Deschout *et al.*, 2014). SPDM allowed the determination of the number of P-glycoprotein efflux transporter molecules present per cell at the blood–brain barrier and showed that they cluster (Huber *et al.*, 2012). Moreover, by two-colour PALM, quantitative information on the tumour necrosis factor receptor 1 cluster sizes and copy numbers were obtained in HeLa cells (Fricke *et al.*, 2014).

Super-resolution microscopy also enables assessment of the third dimension in biological specimens (Hajj *et al.*, 2014). 3D-PALM was used to measure the spatial distribution of H2B nucleosomes to determine the degree of chromatin condensation in human osteosarcoma cells (Recamier *et al.*, 2014), and reflected light-sheet super-resolution microscopy was applied to quantify in 3D the absolute number of RNAPII molecules in the same cells (Zhao *et al.*, 2014).

The combination of multicolour structured illumination microscopy (SIM) and localization microscopy was applied to map in three dimensions Bld12p/CrSAS-6 molecules in basal bodies of *Chlamydomonas* (Rossberger *et al.*, 2013; Hamel *et al.*, 2014).

In *Arabidopsis*, SIM has already been used successfully to identify fluorescent structures in various tissues (Bell *et al.*, 2013; Komis *et al.*, 2014) and sorted nuclei (Schubert *et al.*, 2013; Schubert, 2014).

Here for the first time the numbers of RNAPII molecules in differentiated endopolyploid plant nuclei were determined by applying 3D-PALM. It is believed that this approach is well suited to approximate absolute numbers better than other technologies. In single- and two-colour experiments, it is shown that RNAPII molecules are dispersed evenly within euchromatin, but that they may also form clusters. In addition, it is confirmed that transcription increases with a higher degree of endopolyploidy.

## Materials and methods

### Preparation of nuclei and immunolocalization

Differentiated rosette leaves from 4-week-old *Arabidopsis thaliana* (L.) Heynh. (Columbia) plants grown under short-day conditions (8h light/16h darkness) were fixed for 20min under vacuum in 4% formaldehyde in TRIS buffer (pH 7.5) and homogenized in TRIS buffer. Suspended nuclei were stained with 4′,6-diamidino-2-phenylindole (DAPI) (1 μg ml^–1^) and flow-sorted according to their ploidy level using a FACS Aria flow cytometer (BD Bioscience) onto 22×22mm high precision coverslips (Marienfeld, Germany) in a drop of buffer containing 100mM TRIS, 50mM KCl, 2mM MgCl_2_, 0.05% Tween, 5% sucrose, then air-dried and used for immunolabelling.

For co-localization and quantification of active and inactive modifications of RNAPII, immunostaining was performed according to Jasencakova *et al.* (2000).

The non-phosphorylated (inactive) enzyme was detected with mouse monoclonal antibody (1:300; Abcam, ab817) and goat anti-mouse Alexa 488 (1:200; Invitrogen) or goat anti-mouse-Cy5 (1:300; Jackson ImmunoResearch).

RNAPIISer5ph (active; phosphorylated at Ser5) was detected with rabbit polyclonal antibody (1:200; Active Motif, 39233) and goat anti-rabbit Alexa488 (1:200; Jackson ImmunoResearch), and RNAPIISer2ph (active; phosphorylated at Ser2) with rat monoclonal antibody (1:200; Millipore, 04-1571) and goat anti-rat Alexa488 (1:200; Jackson ImmunoResearch).

### Structured illumination microscopy (SIM)

To analyse the substructural organization of RNAPII molecules beyond the classical Abbe-Rayleigh limit of ~250nm, SIM was applied that yields a 2-fold improvement in all spatial directions. Coverslips bearing the labelled nuclei were placed into Chamlide™ magnetic chambers (Live Cell Instrument, South Korea) and submerged in phosphate-buffered saline (PBS; pH 7.5) supplemented with 1% β-mercaptoethanol prior to SIM imaging on a Zeiss ELYRA PS.1 microscope (Carl Zeiss Microscopy, Germany) equipped with a Plan-Apochromat 63×1.4 oil objective. Optimal grid sizes for each wavelength were chosen according to the recommendations of the manufacturer. For 3D-SIM, stacks with a step size of 110nm were acquired sequentially for each fluorophore starting with the highest wavelength dye. The centre of the stack was chosen to coincide with the main plain along the axis of the ellipsoidal nuclei to allow the alignment of SIM and PALM images. The correction of chromatic aberrations was performed with the ZEN Channel alignment tool using a template obtained from imaging TetraSpeck fluorescent microspheres (200nm in diameter; InVitroGen) and affine correction. Thus, the corrections achieved a precision of <100nm.

### Photoactivated localization microscopy (PALM)

The same set-up was subsequently used to perform 3D-PALM with the PRLIM (phase ramp localization imaging microscopy) implementation (Baddeley *et al.*, 2011). In general, the EM-CCD gain was set to 200V. To avoid *z*-drift, the Definite Focus of the instrument was used, which kept the *z*-position within 30nm. The focal position was chosen to coincide with the central slice of the SIM stack which corresponded to the main plane along the long axis of the nuclei. The *z* capture range was ~2 μm, which allowed the whole *z* distance of the nuclei to be covered for counting. All dye molecules were transferred into their dark state by using high laser power (~10 kW cm^–2^) of the imaging laser followed by 3D-PALM to record the number and localization of single blinking molecules at a lateral resolution of ~20nm and an axial resolution of ~80nm. PALM two-colour experiments were performed first for the long wavelength dye (Cy5) followed by the short wavelength dye (Alexa488). The PALM experiments continued for one dye until the blinking molecules observed were negligible, which needed ~30 000 frames at an integration time of 20ms. Channels were colour aligned in the ZEN channel alignment tool using a template generated with TetraSpeck microspheres (200nm in diameter). This allowed precise alignment with an error of <1 pixel (corresponding to 20nm).

### Processing and analysis of PALM data

To generate PALM images (vector maps), the PALM processing function of the ZEN software was applied. A multi-emitter model was used to account for overlapping signals. For peak finding, the peak mask size was set to 9 pixels, and the noise filter to 6. For localization of 2D-PALM data, the identified peaks were fitted to a 2D Gaussian function using a theoretical point spread function (PSF), and the localization precision was determined according to the Thompson formula for 2D-PALM (Thompson *et al.*, 2002). For 3D data, the identified peaks were fitted to an experimentally acquired PSF and the precision determined by simulations. The created vector map of localizations was then drift corrected using the model-based approach with a precision of <20nm in the *x*, *y*, and <80 nm in the *z* direction. Next, signals were grouped. Two signals falling within the range of 1 pixel (corresponding to 20nm) were regarded as originating from the same molecule, if the ‘on time’ of the molecule was <5 frames (20ms per frame) and the ‘off time’ not more than 20 frames. The latter two criteria reflect the blinking characteristics of organic dyes.

To visualize the distribution of localized molecules, heat maps (Rainbow look-up tables) were used. Albeit that the intensities are dependent on both localization precision and number of molecules, the latter dominated in the experiments conducted here as the majority of localization precisions ranged in a narrow window between 20nm and 60nm. Thus, a high intensity staining signifies the accumulation of molecules. To assess the extent of such accumulations and their spacing, the analysis was restricted to a central *z*-slice of 110nm which was set as a filter. Contours of the heat map of the central slice were used to approximate such accumulations as circles or ellipses. The radii and axes were used to estimate the dimension of the areas of accumulated molecules and their spacing was defined as the distances of their centres. In this way, three levels of clustering were identified: single molecules, and small and large clusters. Ten 8C nuclei were used to analyse the sizes of the clusters and their distances.

To count molecules, the samples were grouped as described to avoid counting an emitter twice. To be as quantitative as possible, labelling efficiencies must be as high as possible. Antibodies were used that proved to be the most efficient in the experiments conducted herein at a concentration slightly above saturation. Since the nuclei were stained in the presence of excess antibodies, it is estimated that at least 90% of the molecules should be labelled. Another issue is to evaluate how many molecules will be assessed by PALM. Based on PALM acquisition under similar experimental settings, it is possible to be confident of detecting ~95% of the labelled molecules. Such a fraction was estimated by comparing PALM with electron microscopic images of nuclear pore complex proteins of isolated nuclei (Löschberger *et al.*, 2014). Thus it is believed that the maximum error is in the range of <15%.

The mean of RNAPII counts and confidence intervals (95%) of the endopolyploid nuclei were calculated with the SigmaPlot 2000 software. Significance levels were estimated by one-way analysis if variance (ANOVA) testing using the Analysis Software of Microsoft Excel. Differences were considered to be significant at *P*-values <0.01.

### Correlation between SIM and PALM images

Channels of the SIM and PALM images were subsequently aligned with the ZEN software (Carl Zeiss Microscopy, Germany) inbuilt Channel Alignment tool using structural features common in both channels. SIM and PALM images were acquired on the same microscope with the same objective, hence magnifications are identical. Only the pixels size (lateral 80nm for SIM, 20nm for PALM) and sectioning (110nm sectioning for SIM, 40nm for PALM) were different. Prior to alignment, the PALM images were rendered to a precision corresponding to the resolution of SIM; that is, 120nm laterally and 360nm axially. In addition, the pixel size and slice thickness of the PALM image were set to the pixel size and slice thickness of the SIM image. After channel alignment, manual correction with the ZEN channel shift tool was employed. In particuloar, the nuclear envelope and the nucleolus were excellent landmarks for matching the images as they could be easily identified since the nucleolus and the region beyond the nucleus were mainly devoid of staining. Such an alignment approach was within the precision of 1 voxel (corresponding to 80nm lateral and 350nm axial). After alignment, the localization precision of PALM was changed back to Gaus rendering and the pixel size and slices were reduced to the initial values.

## Results

By applying SIM, it has been proven that the relative amounts of RNAPII enzymes in differentiated 2C–32C leaf nuclei of *A. thaliana* proportionally increase with increasing endopolyploidy (Schubert, 2014). To determine the number of RNAPII molecules per nucleus to a better extent and to check whether transcription factories may exist in plants, nuclei were labelled with specific antibodies against active and inactive RNAPII modifications and 3D-PALM was applied in combination with SIM.

**Fig. 1.**
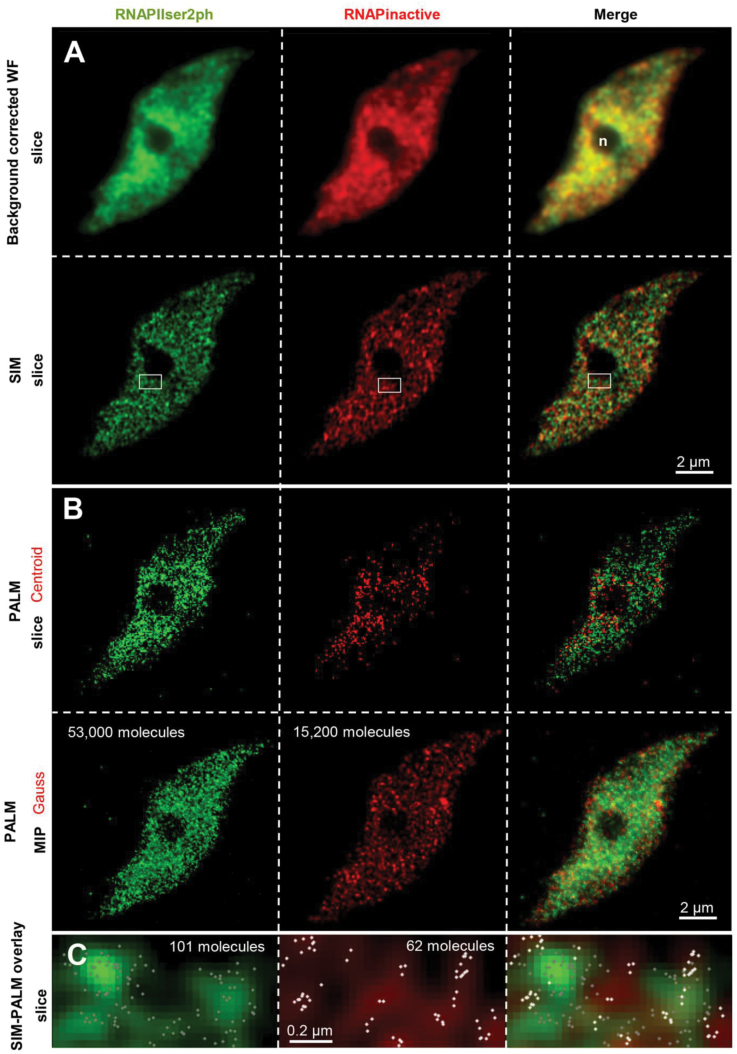
Distribution of inactive (not phosphorylated) and active (phosphorylated at Ser2) RNAPII molecules in an endopolyploid 8C *A. thaliana* leaf nucleus shown after applying background-corrected widefield (WF), SIM, and 3D-PALM. (A) In contrast to WF, SIM clearly shows that both RNAPII modifications form distinct intermingled reticulate structures within the nucleus which are absent from the nucleolus (n). (B) PALM reveals the number of RNAPII molecules (as centroids) in the same optical slice as shown in (A) (upper panel). The lower panel represents Gauss-rendered maximum intensity projections (MIPs) generated from all PALM slices to show the complete number of molecules evident in the nucleus. Thus the higher amount of active (53 000) compared with inactive (15 200) RNAPII molecules becomes obvious. (C) The overlay of the SIM (insets in A) and PALM image indicates the number and distribution of single RNAPII molecules within the structures resolved by SIM. The images show that both active and inactive molecules cluster in distinct regions of the nucleus.

After acquiring SIM images to determine the distribution of the RNAPII enzymes at lateral and axial resolutions of 120nm and 360nm, respectively, reticulate structures for the active and inactive RNAPII variants, which were not detectable by classical wide-field microscopy, became visible (Fig. 1A). Then, most of the molecules were transferred into their dark state by high laser power (~10 kW cm^–2^) followed by 3D-PALM to record the number and localization of single blinking molecules at a lateral resolution of ~20nm and an axial resolution of ~80nm (Fig. 1B, C). When molecules were rendered to the obtained localization precision, both active and inactive RNAPII variants distributed uniformly throughout the nucleus, with the exception of the nucleolus that was devoid of RNAPII (Fig. 1B; Supplementary Fig. S1 available at *JXB* online). The combination of SIM with PALM and the simultaneous labelling of different RNAPII variants with the fluorophores Alexa488 and Cy5 in the same nucleus allowed single molecules to be localized within the reticulate structures (Fig. 1C). No co-localization of active and inactive RNAPII appeared, suggesting that both variants occupy distinct areas within euchromatin. This finding is quite interesting because theoretically it could be expected that partially phosphorylated RNAPII molecules would also be identified by both antibody variants.

**Fig. 2.**
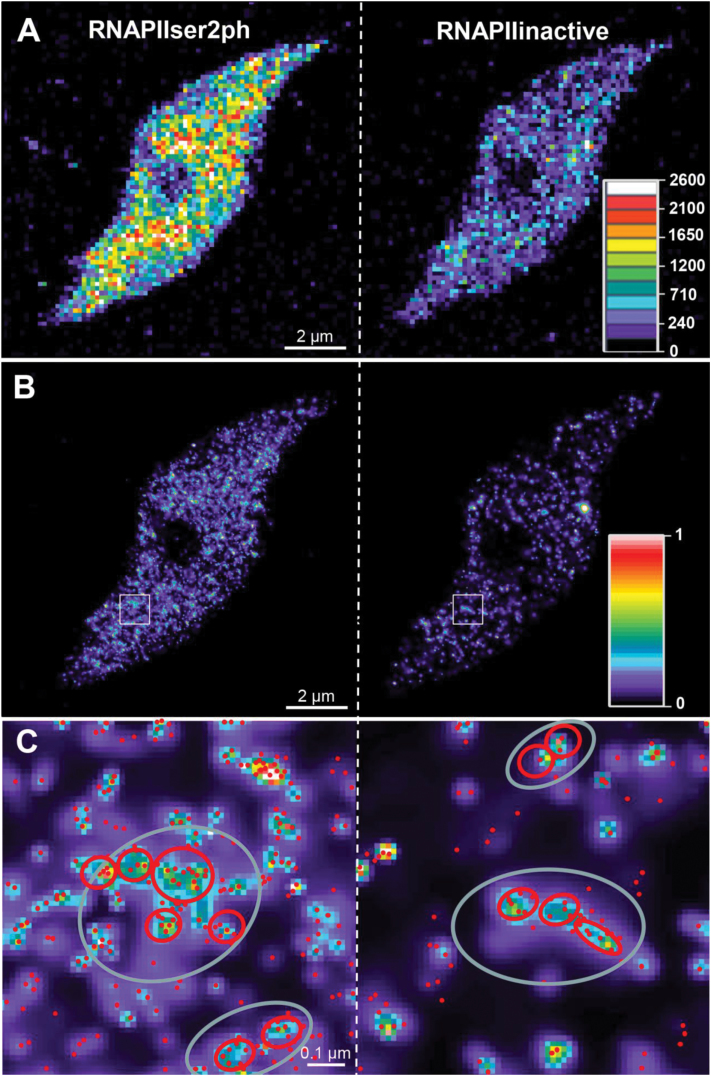
Distribution of inactive and active RNAPII molecules in an 8C *A. thaliana* nucleus (Nucleus 1 in Supplementary Table S1C at *JXB* online) recorded by 3D-PALM. (A) Density map of inactive (not phosphorylated) and active (phosphorylated at Ser2) RNAPII in a 110nm slice. The density colour code represents molecules per μm^3^. (B) Gauss-rendered (displayed in a heat map; 1=100% intensity) 3D-PALM images to emphasize intensity differences. (C) Enlarged image of the area boxed in (B) showing the intensity distribution (see the scale in B) with localized molecules (red dots) within the heat map. Short distance clusters are encircled in red, long distance clusters in grey.

**Fig. 3.**
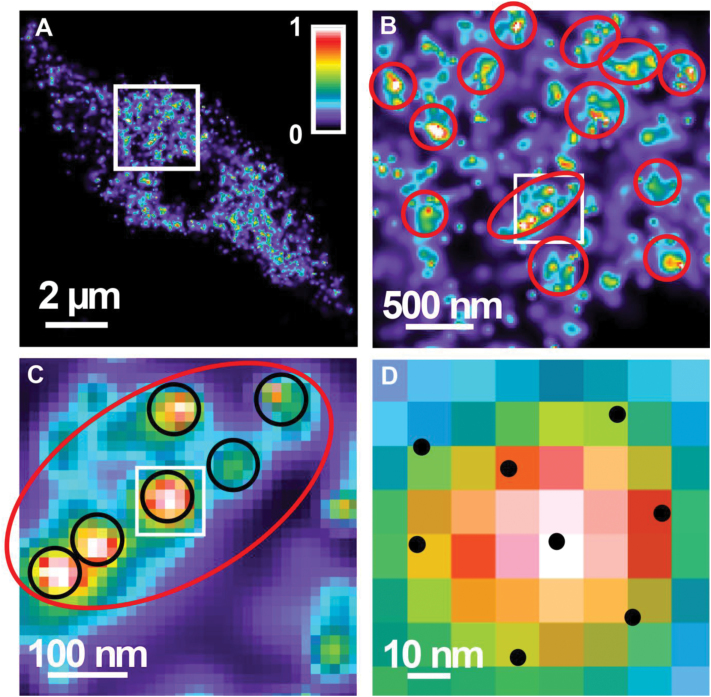
Distribution and aggregation of RNAPIISer2ph (false coloured in a heat map with the intensity colour code indicated). (A) Distribution in a 110nm slice of an 8C nucleus. (B) Enlarged image of the boxed area in (A). Red circle and ellipses represent large clusters. (C) Enlarged image of the boxed area in (B). The red ellipse and the black circles represent large and small clusters, respectively. (D) Enlarged image of the boxed area in (C). Black dots represent single molecules.

**Fig. 4.**
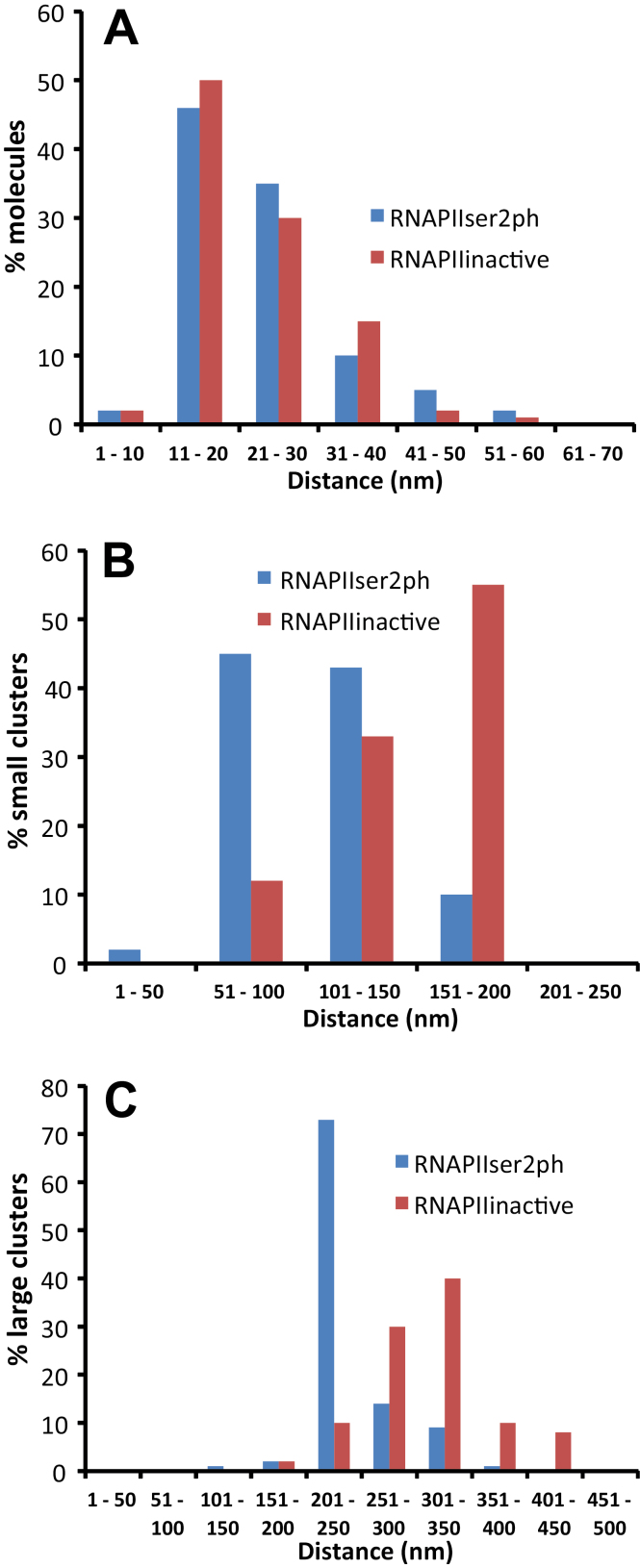
Distances between single molecules (A), small (B), and large clusters (C).

Due to the presence of more molecules, the active RNAPII showed a higher global density than the inactive variant (Fig. 2A). However, both variants exhibited similar cluster behaviour with regularly occurring sites of higher accumulation (Figs 2B, C, 3). The distance between single RNAPII molecules within small clusters ranged between 20nm and 40nm for both active and inactive variants (Fig. 4A). In addition, there are regions in which the small clusters further accumulate at distances between 50nm and 150nm, and 100nm and 200nm (large clusters) for active and inactive RNAPII, respectively (Fig. 4B). The distances between the large clusters amounted to 200–300nm for active and 200–400nm for inactive RNAPII (Fig. 4C). Thus, in addition to a global dispersion of both RNAPII variants within euchromatin, RNAPII molecules may also aggregate at two different levels. Despite a similar mean single molecule distance of 27nm, the active RNAPII molecules aggregate more densely in small and large distance clusters than the inactive molecules. Small and large clusters correspond to the size known for animal transcription factories. The diameters of small clusters ranged between 60nm and 90nm, that of round large clusters between 250nm and 400nm, and that of large ellipsoid clusters between 120–250nm and 250–700nm, respectively. On average, in the same euchromatic area (measured in a 110nm slice of 10 different 8C nuclei), 50–75 large active RNAPII clusters (containing 2–8 small clusters) correspond to 25–40 large inactive RNAPII clusters (containing 2–5 small clusters). The mean small cluster distance was 130nm and 170nm and for large clusters it was 265nm and 335nm for active and inactive RNAPII, respectively.

**Fig. 5.**
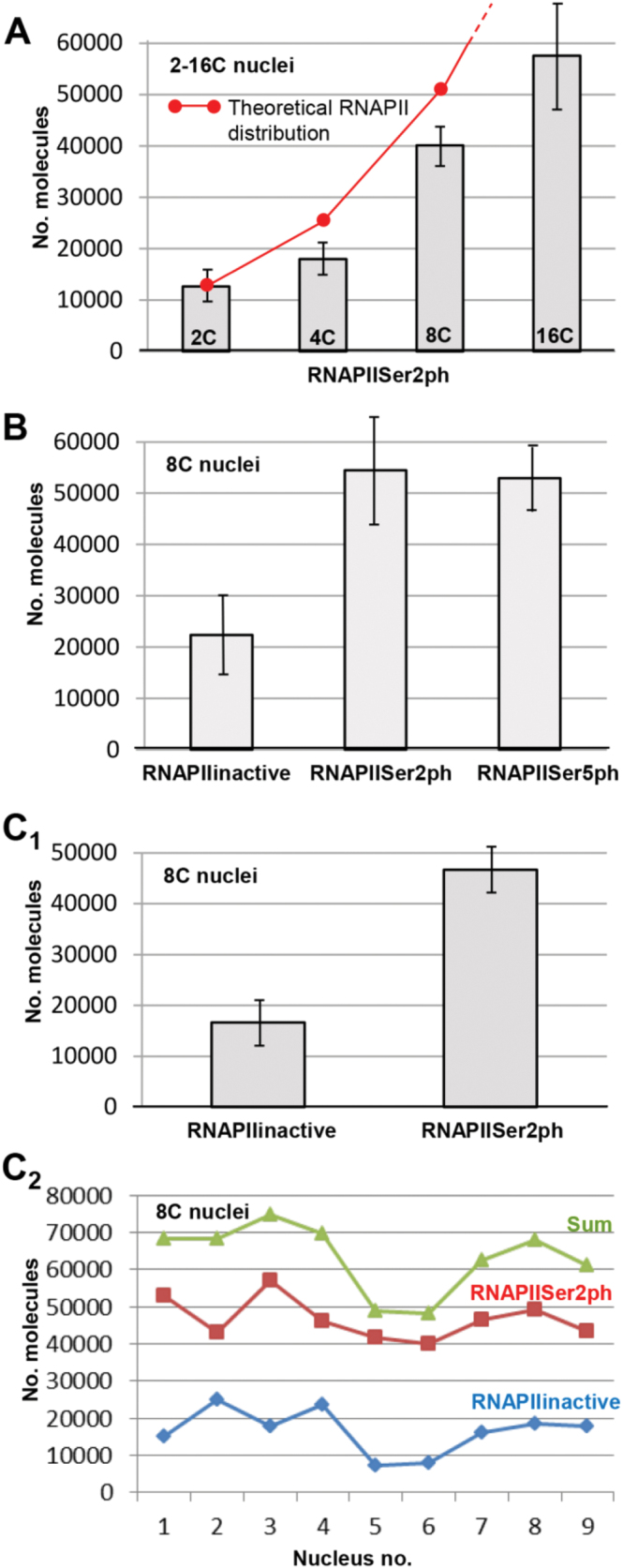
Mean (A, B, C_1_) and individual (C_2_) numbers of RNAPII molecules in differentiated *A. thaliana* leaf nuclei (95% confidence intervals are indicated; for statistics, see Supplementary Table S1 at *JXB* online). (A) Mean number of RNAPIISer2ph in 2C–16C nuclei. The theoretical RNAPII distribution (red dots) indicates the values expected in the case of exact enzyme duplication during endoreduplication. (B) Mean number of inactive and active (RNAPIISer2ph, RNAPIISer5ph) RNAPII molecules in 8C nuclei. (C_1_) Mean number of inactive RNAPII and RNAPIISer2ph in simultaneously labelled 8C nuclei. (C_2_) Numbers of inactive and active (RNAPIISer2ph) RNAPII molecules in nine different nuclei simultaneously labelled with specific antibodies.

3D-PALM also allowed the estimation of the copy numbers of active and inactive RNAPII within the whole nucleus. In differentiated 2C and endopolyploid 4C–16C leaf nuclei, the mean number of RNAPIISer2ph increased and ranged between ~13 000 in 2C and ~58 000 in 16C nuclei (Fig. 5A; Supplementary Table S1A at *JXB* online), thus confirming the data obtained by Schubert (2014) based on signal intensity measurements in maximum intensity projections of SIM image stacks. However, the molecule numbers did not increase proportionally as expected theoretically in the case of exact enzyme duplication during endoreduplication and as has been found by Schubert (2014).

In a second experiment in 8C nuclei, the mean number of both active (RNAPIISer2ph and RNAPIISer5ph) variants amounted to ~53 000 and thus was clearly higher than the number of the inactive molecules (~22 000; Fig. 5B; Supplementary Table 1B at *JXB* online). In different experiments it became obvious that active RNAPII is ~2.5 times as abundant as the inactive variant (Fig. 5B, C_1_). In contrast, the signal intensity measurements by Schubert (2014) indicated similar amounts of active and inactive RNAPII enzymes.

To test whether an increased transcriptional activity (indicated by a higher number of active RNAPII molecules) induces the decrease of already available inactive molecules, 8C nuclei were labelled simultaneously with antibodies against inactive and active (phosphorylated at Ser2) RNAPII, and 3D-PALM was applied (Figs 1, 5 C_1_). Considering the amount of both RNAPII variants in single nuclei, it seems that such a correlation does not exist because in most nuclei a low or high number of inactive RNAPII molecules is accompanied by a corresponding active molecule number (Fig. 5C_2_; Supplementary Table S1C at *JXB* online).

In summary, it is concluded that (i) the reticulate structures formed by the different RNAPII modifications and identified by SIM contain, as proven by PALM, several globally mainly dispersed single molecules which may cluster within a size known from animal transcription factories; (ii) RNAPII quantification by 3D-PALM is more reliable than signal intensity measurements in maximum intensity projections generated from SIM image stacks; and (iii) the 3D-PALM measurements confirm that the number of RNAPII molecules increases with the degree of endopolyploidy.

## Discussion

### 3D-PALM reliably counts and co-localizes single molecules in isolated nuclei

In this report, a version of 3D-PALM called phase ramp imaging localization microscopy (PRILM), a method that among other 3D technologies provides the largest capture range in the axial direction per measurement position (Baddeley *et al.*, 2010), was applied. Due to the outstanding signal-to-noise ratio achieved by the staining procedure on isolated nuclei and their flattening on the coverslips, a *z* dimension of ~2 μm was obtained, namely flat enough to be fully covered in their *z*-extension by PRILM during only one measurement step. Thus, it was possible to avoid multiple measurements at different *z*-planes that potentially can affect the counting efficiency.

Regardless of the approach, the true number of molecules depends on different factors such as labelling efficiency, molecule density, and dye properties (Dempsey *et al.*, 2011; Durisic *et al.*, 2014). To ensure that as many molecules as possible were labelled antibodies were chosen which provided the best labelling efficiencies at concentrations above saturation, and dyes with high quantum yields were used. As internal controls, the secondary antibodies were swapped between the different colours to show that they had no influence on the overall counting efficiencies. To reduce the possibility of counting a molecule twice, the events were grouped in such a way that those lying within the localization precision were regarded as originating from the same molecule. Recordings were made until no significant blinking appeared. In all three experiments using different nuclei from different preparations (Fig. 5; Supplementary Table S1 at *JXB* online), similar mean numbers of RNAPII molecules per nucleus were obtained (compare RNAPIIinactive and RNAPIISer2ph), indicating the reliability of the 3D-PALM measurements.

Although a constant increase in gene copy numbers is accompanied by an increasing endopolyploidy level, the theoretically expected doubling of RNAPII molecules was not observed (Fig. 5A). The increases varied between 1.38- and 2.2-fold, with an average increase of 1.68-fold. This might reflect counting inaccuracies. For example, when comparing the counts of three different preparations of 8C nuclei, variations by a factor of 1.3–1.8 were observed (compare RNAPIISer2ph in Fig. 5A, B, and C_1_). On the other hand, with the doubling of the gene copy numbers, other cellular factors could also possibly limit a parallel doubling of RNAPII molecules.

Despite these considerations, the 3D-PALM counting allowed a more accurate estimate of the RNAPII amount to be obtained than in a previous intensity-based approach (Schubert, 2014). Intensity-based methods lack absolute quantitative information as higher intensities could be caused by more molecules less densely arranged or fewer molecules with a denser arrangement. PALM allows unambiguous discrimination between density and amount of molecules by accurately localizing and counting them. Active RNAPII seems to be more tightly arranged probably due to its ~3-fold higher abundance than the inactive variant (Fig. 2A). If RNAPII molecules are more densely packed, the probability of fluorophore quenching might be higher.

### RNAPII appears globally dispersed, but also aggregates within euchromatin

In mammals, genes with similar functions may be co-localized and co-expressed in postulated distinct transcription factories via out-looping from chromatin (Iborra *et al.*, 1996; Edelman and Fraser, 2012; Rieder *et al.*, 2012; Papantonis and Cook, 2013), as has been proven, for example, for active immunoglobulin genes from three different mouse chromosomes (Park *et al.*, 2014). In contrast, in a recent report, it has been found in human osteosarcoma cells by applying reflected light-sheet super-resolution microscopy that >70% of the transcription foci originate from single RNAPII molecules with a mean distance of ~230nm (Zhao *et al.*, 2014). In the same report, no clustering between RNAPII molecules was detected within the range of ~40–200nm (mean=130nm), which was predicted for transcription factories (Iborra *et al.*, 1996; Eskiw and Fraser, 2011). Thus, the majority of the RNAPII molecules may exist in a solitary fashion inside the mammalian nucleus, and Zhao *et al.* (2014) conclude that the idea of a co-ordinated transcription of mammalian genes in prevalently existing transcription factories needs to be revisited. These findings deviate from the previous results because, in addition to a global RNAPII dispersion, an aggregation of RNAPII molecules within a size predicted for animal and human transcription factories was also found. Thus it is argued that in plant nuclei transcription factories may exist and that they are homogeneously dispersed within euchromatin. The slightly higher distances of inactive RNAPII may be due to its lower abundance compared with active RNAPII.

Based on a global transcription factor localization analysis within a single human cell type, Yan *et al.* (2013) described the majority of these factors to be arranged in clusters also containing cohesins. They may be important for the re-establishment of transcription factor clusters after DNA replication and for chromatin condensation and thus for maintaining the transcriptional memory of dividing cells.

Markaki *et al.* (2010) desribe a network of channels and lacunas, called the interchromatin compartment, throughout mammalian nuclei which contain decondensed chromatin. In this region, nascent DNA, nascent RNA, RNAPII, and histone modifications for transcriptionally active chromatin, are highly enriched.

Similarly, the distribution of RNAPII as resolved by SIM was identified in a network-like manner within euchromatin of rye and *Arabidopsis*. RNAPII was absent from nucleoli and heterochromatin (Schubert, 2014). The inactive form (not phosphorylated) showed more distinct signals than the active RNAPII forms (phoshorylated at Ser2 and Ser5, respectively). In accordance with this, it is shown here by 3D-PALM that within these reticulate structures single molecules are dispersed and additionally that both the inactive and the active RNAPII molecules may aggregate, but not active and inactive RNAPII together.

Interestingly, the structural maintenance of chromosome (SMC) cohesin and condensin complex subunits SMC3 and CAP-D3, respectively, show a reticulate distribution within euchromatin of differentiated endopolyploid *Arabidopsis* nuclei (Schubert *et al.*, 2013) similar to that of RNAPII. In these nuclei, single chromatid segments are mainly not cohesive within euchromatic chromosome territories, possibly making multiple gene copies accessible for transcription (Schubert *et al.*, 2012). Despite a stable global interphase chromatin organization, intra- and interchromosomal associations indicative for co-ordinated transcription may appear, but more seldom at interstitial euchromatin segments than at subtelomeres and pericentromeres (Grob *et al.*, 2013; Schubert *et al.*, 2014; Feng *et al.*, 2014). According to data found in Hi-C experiments, epigenetic marks of active chromatin did not show a co-localization with highly associated chromatin segments in *A. thaliana*, suggesting a lack of clustering of the most actively transcribed genes (Feng *et al.*, 2014). This observation is supported by the finding that interstitial euchromatin segments containing highly co-expressed genes do not associate more often than those containing genes co-expressed at a low level (Schubert *et al.*, 2014). Thus, it seems that in *Arabidopsis* most of the transcriptional activity is not only localized in potential transcription factories but is also homogeneously distributed within euchromatin, indicating that most of the *Arabidopsis* genes are not induced by co-expression as described for yeast, *Drosophila*, and mammals (Osborne et al., 2004, 2007; Brown *et al.*, 2006, 2008; Tanizawa *et al.*, 2010; Gibcus and Dekker, 2012, 2013; Hou and Corces, 2012). Similarly, Abranches *et al.* (1998) found a uniform distribution of transcription sites throughout the nucleoplasm of wheat.

**Fig. 6.**
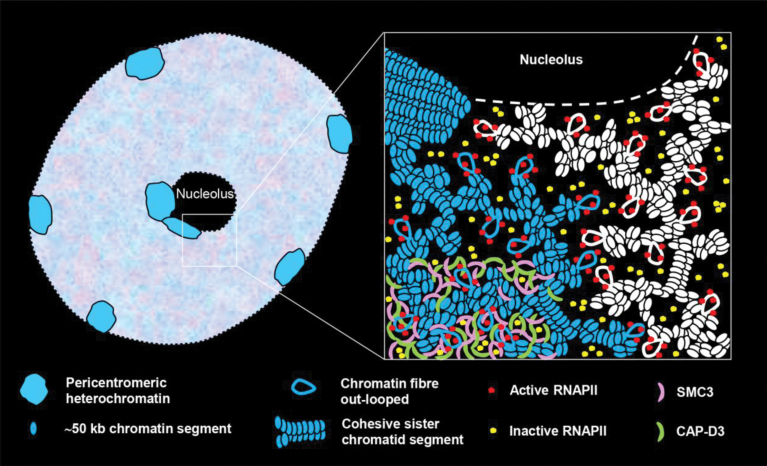
Model of the arrangement of RNAPII and SMC protein complex subunits (SMC3 and CAP-D3) within euchromatin of interphase plant nuclei (exemplified for a 4C *A. thaliana* leaf nucleus). The proteins are absent from heterochromatin and the nucleolus. The inset shows, at higher magnification, the chromatin arrangement of two different adjacent chromosome arm territories (blue and white). The heterochromatin is highly condensed. The euchromatin of the sister chromatids is composed of ~50kb chromatin segments which cluster further. Sister chromatid segments may be cohesive or separated. Chromatin fibres may emanate where RNAPII molecules cluster and become activated in potential transcription factories. Inactive RNAPII enzymes, mostly also aggregating, distribute within euchromatin. The SMC3 cohesin and CAP-D3 condensin subunits are homogeneously distributed (shown in the bottom left corner of the inset) possibly responsible for maintaining a flexible euchromatin organization.

Based on previous findings and those presented here regarding the *Arabidopsis* interphase chromatin arrangement (Schubert *et al.*, 2012), and the distribution of cohesin and condensin proteins (Schubert *et al.*, 2013) and RNAPII molecules (Schubert, 2014), a model of the organization of endopolyploid plant interphase nuclei is proposed in which SMC proteins are involved in maintaining a euchromatin structure allowing flexible dispersed transcription (Fig. 6).

### How many genes are transcribed in a nucleus?

In contrast to temporally induced genes, from prokaryotes to mammals, due to stochastic fluctuations in transcription, the expression of constitutive genes encoding essential subunits of protein complexes can vary by at least ~50% among genetically identical cells. Nevertheless, the resulting proteins show a similar abundance and less variability (Golding *et al.*, 2005; Sigal *et al.*, 2006; Raj et al., 2006, 2010; Zenklusen *et al.*, 2008; Cohen *et al.*, 2009; Pare *et al.*, 2009; Taniguchi *et al.*, 2010; Reiter *et al.*, 2011; Gandhi *et al.*, 2011). In addition, the gene activation causes burst-like expression of all genes within a larger chromatin segment (Raj *et al.*, 2006). This supports the idea that transcription is intrinsically stochastic, providing a flexibility important for cells to respond to changing environments and sudden stress, and to provide a cell population heterogeneity needed during cellular differentiation and development (Kaern *et al.*, 2005; Li and Xie, 2011; Velez-Bermudez and Schmidt, 2014). Little *et al.* (2013) found that, despite fluctuating transcription, the accumulation of mRNAs is similar across essential *Drosophila* embryo patterning genes generating precise protein distributions. Furthermore, these authors suggest that fluctuations in mRNA production are context independent and are a fundamental characteristic of transcription, thus resolving the apparent paradox between stochastic transcription and developmental precision.

Stochastic fluctuations in transcription may explain the high variability in the numbers of RNAPII molecules found between individual nuclei of the same ploidy level (Supplementary Table S1 at *JXB* online). Compared with the observed difference of ~50% regarding active RNAPIIs, it even reached as much as 70% for inactive RNAPII. However, the heterogeneity of the number of RNAPII molecules may also be caused by the different leaf cell types from where the sorted nuclei originate.

It has been suggested that ~75% of the human genome is associated with transcriptional activity (Djebali *et al.*, 2012). A lower amount was determined for *A. thaliana* where the global level of expression depends on growth conditions (laboratory controlled or field conditions) and the accession analysed (Schmid *et al.*, 2005; Kliebenstein *et al.*, 2006; Richards *et al.*, 2012). In the shoot, 45–61% of genes were expressed at each time point under field conditions (Richards *et al.*, 2012).

*Arabidopsis thaliana* has 27 379 genes (Arabidopsis Genome Initiative, 2000) and for rye ~26 000 genes are expected, as found for the closely related barley (International Barley Genome Sequencing Consortium, 2012). This may explain the similar amounts of active as well as inactive RNAPII found by SIM signal intensity measurements in meristematic nuclei of both species (Schubert, 2014).

Here it is confirmd by single molecule localization that the amount of RNAPII increases with endopolyploidy in plants (Bourdon *et al.*, 2012; Schubert, 2014). Thus, increasing the transcriptional activity of cells and tissues seems to be an important function of endopolyploidy.

Assuming that active RNAPII enzymes are phosphorylated only either at Ser2 or at Ser5 and not in parallel, the mean sum of RNAPII molecules can be calculated (22340inactive+54480Ser2ph+52900Ser5ph; Fig. 5B; Supplementary Table S1B at *JXB* online) and thus amounts to ~107 000 active and in total to ~130 000 molecules per 8C nucleus.

By reflected light-sheet super-resolution microscopy, Zhao *et al.* (2014) determined that there were ~80 200±8800 active RNAPII molecules within the transcription foci of human osteosarcoma cell nuclei, a number in agreement with that previously reported in human nuclei based on other methods (Sugden and Keller, 1973; Jackson et al., 1998, 2000; Pombo *et al.*, 1999).

Assuming that these human nuclei are diploid and that half of them are in G_1_ phase (containing two unreplicated chromatids, i.e. two copies per gene) and the others are in G_2_ (containing four replicated chromatids, i.e. four copies per gene) and regarding that the human genome contains 20 687 protein-coding genes (Pennisi 2012), the mean gene copy number per nucleus should be 62 061 (20 687×2+20 687×4=124 122÷2). Thus ~1.3 (80 200÷62 061) active RNAPII enzymes are present per gene copy.

In differentiated 4C *A. thaliana* nuclei containing four chromatids, 109 516 (27 379×4) gene copies are available. In addition, ~36 000 (~18 000 RNAPIISer2ph+~18 000 expected RNAPIISer5ph because the amount is similar in 8C nuclei) (Fig. 5B; Table 1) active RNAPII molecules should be present per nucleus. These are fewer (36 000÷109 516=0.3) active RNAPII molecules per gene copy than found in cultured and cycling human cells, probably caused by the differentiated status of these plant cells. Fewer RNAPII molecules per gene copy is in accordance with the observation that more human genes are associated with transcriptional activity than those of *A. thaliana* (see above). Interestingly, with 0.3–0.5 RNAPII molecules per gene copy, the ratio is similar at all four ploidy levels in *A. thaliana*.

**Table 1.** *Number of active RNAPIISer2ph and RNAPIISer5p molecules in differentiated 2C–16C leaf nuclei determined by 3D-PALM (see Supplementary Table S1A at JXB online) in comparison with the available gene copy number calculated on the basis of the 27 379 genes evident in* A. thaliana *(TAIR9; Arabidopsis Genome Initiative, 2000)*

Ploidy	No. of gene copies	No. of RNAPIISer2ph	No. of RNAPIISer5ph^*a*^	Total active RNAPII molecules	Active RNAPII molecules per gene
2C	54 758	12 630	12 630	25 260	0.5
4C	109 516	17 960	17 960	35 920	0.3
8C	219 032	40 130	40 130	80 260	0.4
16C	438 064	57 590	57 590	115 180	0.3

^*a*^ A similar amount expected as found for RNAPIIphSer2 based on the data shown in Supplementary Table S1.

By two-colour labelling and 3D-PALM, it was observed that in addition to active RNAPIISer2ph molecules responsible for transcriptional elongation, inactive RNAPII molecules are present in these nuclei with a frequency of about half of that of active enzymes. Because the density of active RNAPII on genes depends on the initiation frequency, elongation frequency, and processivity (RNAPII remaining on the template after each catalytic event) (Ehrensberger *et al.*. 2013), it is difficult to calculate the number of transcribed genes per nucleus. However, assuming that only one RNAPII Ser2ph molecule responsible for transcriptional elongation would be associated with only one gene, as mostly found in *Drosophila*, mouse, and human nuclei (Laird and Chooi, 1976; McKnight and Miller, 1976; Fakan *et al.*, 1986; Jackson *et al.*, 1998), then every second to third gene would be active in 2C–16C *A. thaliana* leaf nuclei (Table 1). Due to the presence of mainly non-cohesive chromatids in highly endopolyploid *A. thaliana* nuclei (Schubert *et al.*, 2012), the resulting ‘open chromatin’ structure (Zhang *et al.*, 2014) may allow the parallel transcription of multiple gene copies by RNAPII as demonstrated at the four well-separated chromatids in *Drosophila* embryo nuclei (Little *et al.*, 2013). Thus, assuming that ~50% of the different genes are active in *A. thaliana* (see above), on average approximately one copy of the same gene would be transcriptionally active in 2C–16C nuclei. However, it could also be possible that less than the half of the genome of endopolyploid nuclei is transcribed, but instead in parallel more gene copies per gene (e.g. up to 16 in 16C nuclei) are transcribed in differentiated endopolyploid nuclei.

## Supplementary data

Supplementary data are available at JXB online.

Figure S1. Gauss rendered PALM image of RNAPIISer2ph localized within reticulate structures acquired by SIM.

Table S1. RNAPII amount in differentiated *A. thaliana* leaf nuclei.

Supplementary Data

## References

[CIT0001] Abranches R Beven AF Aragon-Alcaide L Shaw PJ 1998 Transcription sites are not correlated with chromosome territories in wheat nuclei. Journal of Cell Biology 143, 5–12. 9763416 10.1083/jcb.143.1.5 PMC2132808

[CIT0002] Arabidopsis Genome Initiative. 2000 Analysis of the genome sequence of the flowering plant Arabidopsis thaliana. Nature 408, 796–815. 11130711 10.1038/35048692

[CIT0003] Baddeley D Chagin VO Schermelleh L 2010 Measurement of replication structures at the nanometer scale using super-resolution light microscopy. Nucleic Acids Research 38, e8. 19864256 10.1093/nar/gkp901 PMC2811013

[CIT0004] Baddeley D Crossman D Rossberger S Cheyne JE Montgomery JM Jayasinghe ID Cremer C Cannell MB Soeller C 2011 4D super-resolution microscopy with conventional fluorophores and single wavelength excitation in optically thick cells and tissues. PLoS One 6, e20645. 21655189 10.1371/journal.pone.0020645 PMC3105105

[CIT0005] Bell K Mitchell S Paultre D Posch M Oparka K 2013 Correlative imaging of fluorescent proteins in resin-embedded plant material. Plant Physiology 161, 1595–1603. 23457228 10.1104/pp.112.212365 PMC3613441

[CIT0006] Bourdon M Pirrello J Cheniclet C 2012 Evidence for karyoplasmic homeostasis during endoreduplication and a ploidy-dependent increase in gene transcription during tomato fruit growth. Development 139, 3817–3826. 22991446 10.1242/dev.084053

[CIT0007] Brown JM Green J das Neves RP 2008 Association between active genes occurs at nuclear speckles and is modulated by chromatin environment. Journal of Cell Biology 182, 1083–1097. 18809724 10.1083/jcb.200803174 PMC2542471

[CIT0008] Brown JM Leach J Reittie JE Atzberger A Lee-Prudhoe J Wood WG Higgs DR Iborra FJ Buckle VJ 2006 Coregulated human globin genes are frequently in spatial proximity when active. Journal of Cell Biology 172, 177–187. 16418531 10.1083/jcb.200507073 PMC2063548

[CIT0009] Chakalova L Fraser P 2010 Organization of transcription. Cold Spring Harbor Perspectives in Biology 2, a000729. 20668006 10.1101/cshperspect.a000729 PMC2926752

[CIT0010] Cohen AA Kalisky T Mayo A 2009 Protein dynamics in individual human cells: experiment and theory. PLoS One 4, e4901. 19381343 10.1371/journal.pone.0004901 PMC2668709

[CIT0011] Dempsey GT Vaughan JC Chen KH Bates M Zhuang X 2011 Evaluation of fluorophores for optimal performance in localization-based super-resolution imaging. Nature Methods 8, 1027–1036. 22056676 10.1038/nmeth.1768 PMC3272503

[CIT0012] Deschout H Cella Zanacchi F Mlodzianoski M Diaspro A Bewersdorf J Hess ST Braeckmans K 2014 Precisely and accurately localizing single emitters in fluorescence microscopy. Nature Methods 11, 253–266. 24577276 10.1038/nmeth.2843

[CIT0013] Djebali S Davis CA Merkel A 2012 Landscape of transcription in human cells. Nature 489, 101–108. 22955620 10.1038/nature11233 PMC3684276

[CIT0014] Durisic N Cuervo LL Lakadamyali M 2014 Quantitative super-resolution microscopy: pitfalls and strategies for image analysis. Current Opinion in Chemical Biology 20, 22–28. 24793374 10.1016/j.cbpa.2014.04.005

[CIT0015] Edelman LB Fraser P 2012 Transcription factories: genetic programming in three dimensions. Current Opinion in Genetics and Development 22, 110–114. 22365496 10.1016/j.gde.2012.01.010

[CIT0016] Egloff S Murphy S 2008 Cracking the RNA polymerase II CTD code. Trends in Genetics 24, 280–288. 18457900 10.1016/j.tig.2008.03.008

[CIT0017] Ehrensberger AH Kelly GP Svejstrup JQ 2013 Mechanistic interpretation of promoter-proximal peaks and RNAPII density maps. Cell 154, 713–715. 23953103 10.1016/j.cell.2013.07.032

[CIT0018] Eskiw CH Fraser P 2011 Ultrastructural study of transcription factories in mouse erythroblasts. Journal of Cell Science 124, 3676–3683. 22045738 10.1242/jcs.087981 PMC3215576

[CIT0019] Eskiw CH Rapp A Carter DR Cook PR 2008 RNA polymerase II activity is located on the surface of protein-rich transcription factories. Journal of Cell Science 121, 1999–2007. 18495842 10.1242/jcs.027250

[CIT0020] Fakan S Leser G Martin TE 1986 Immunoelectron microscope visualization of nuclear ribonucleoprotein antigens within spread transcription complexes. Journal of Cell Biology 103, 1153–1157. 2945824 10.1083/jcb.103.4.1153 PMC2114363

[CIT0021] Fay FS Taneja KL Shenoy S Lifshitz L Singer RH 1997 Quantitative digital analysis of diffuse and concentrated nuclear distributions of nascent transcripts, SC35 and poly(A). Experimental Cell Research 231, 27–37. 9056409 10.1006/excr.1996.3460

[CIT0022] Feng S Cokus SJ Schubert V Zhai J Pellegrini M Jacobsen SE 2014 Genome-wide Hi-C analyses in wild-type and mutants reveal high-resolution chromatin interactions in Arabidopsis. Molecular Cell 55, 694–707. 25132175 10.1016/j.molcel.2014.07.008 PMC4347903

[CIT0023] Ferrai C Xie SQ Luraghi P Munari D Ramirez F Branco MR Pombo A Crippa MP 2010 Poised transcription factories prime silent uPA gene prior to activation. PLoS Biology 8, e1000270. 20052287 10.1371/journal.pbio.1000270 PMC2797137

[CIT0024] Fricke F Malkusch S Wangorsch G Greiner JF Kaltschmidt B Kaltschmidt C Widera D Dandekar T Heilemann M 2014 Quantitative single-molecule localization microscopy combined with rule-based modeling reveals ligand-induced TNF-R1 reorganization toward higher-order oligomers. Histochemistry and Cell Biology 142, 91–101. 24519400 10.1007/s00418-014-1195-0

[CIT0025] Gandhi SJ Zenklusen D Lionnet T Singer RH 2011 Transcription of functionally related constitutive genes is not coordinated. Nature Structural and Molecular Biology 18, 27–34. 10.1038/nsmb.1934 PMC3058351 21131977

[CIT0026] Gibcus JH Dekker J 2012 The context of gene expression regulation. F1000 Biology Reports 4, 8. 22500194 10.3410/B4-8 PMC3318259

[CIT0027] Gibcus JH Dekker J 2013 The hierarchy of the 3D genome. Molecular Cell 49, 773–782. 23473598 10.1016/j.molcel.2013.02.011 PMC3741673

[CIT0028] Golding I Paulsson J Zawilski SM Cox EC 2005 Real-time kinetics of gene activity in individual bacteria. Cell 123, 1025–1036. 16360033 10.1016/j.cell.2005.09.031

[CIT0029] Grande MA van der Kraan I de Jong L van Driel R 1997 Nuclear distribution of transcription factors in relation to sites of transcription and RNA polymerase II. Journal of Cell Science 110, 1781–1791. 9264465 10.1242/jcs.110.15.1781

[CIT0030] Grob S Schmid MW Luedtke NW Wicker T Grossniklaus U 2013 Characterization of chromosomal architecture in Arabidopsis by chromosome conformation capture. Genome Biology 14, R129. 24267747 10.1186/gb-2013-14-11-r129 PMC4053840

[CIT0031] Hajheidari M Koncz C Eick D 2013 Emerging roles for RNA polymerase II CTD in Arabidopsis. Trends in Plant Science 18, 633–643. 23910452 10.1016/j.tplants.2013.07.001

[CIT0032] Hajj B El Beheiry M Izeddin I Darzacq X Dahan M 2014 Accessing the third dimension in localization-based super-resolution microscopy. Physical Chemistry Chemical Physiucs 16, 16340–16348. 10.1039/c4cp01380h 24901106

[CIT0033] Hamel V Guichhard P Fournier M Guiet R Flückiger I Seitz A Gönczy P 2014 Correlative multicolor 3D SIM and STORM microscopy. Biomedical Optics Express 5, 3326–3336. 25360353 10.1364/BOE.5.003326 PMC4206305

[CIT0034] Hengartner CJ Myer VE Liao SM Wilson CJ Koh SS Young RA 1998 Temporal regulation of RNA polymerase II by Srb10 and Kin28 cyclin-dependent kinases. Molecular Cell 2, 43–53. 9702190 10.1016/s1097-2765(00)80112-4

[CIT0035] Hirose Y Ohkuma Y 2007 Phosphorylation of the C-terminal domain of RNA polymerase II plays central roles in the integrated events of eucaryotic gene expression. Journal of Biochemistry 141, 601–608. 17405796 10.1093/jb/mvm090

[CIT0036] Hou C Corces VG 2012 Throwing transcription for a loop: expression of the genome in the 3D nucleus. Chromosoma 121, 107–116. 22094989 10.1007/s00412-011-0352-7 PMC3343363

[CIT0037] Huber O Brunner A Maier P Kaufmann R Couraud PO Cremer C Fricker G 2012 Localization microscopy (SPDM) reveals clustered formations of P-glycoprotein in a human blood–brain barrier model. PLoS One 7, e44776. 22984556 10.1371/journal.pone.0044776 PMC3440331

[CIT0038] Iborra FJ Pombo A Jackson DA Cook PR 1996 Active RNA polymerases are localized within discrete transcription ‘factories’ in human nuclei. Journal of Cell Science 109, 1427–1436. 8799830 10.1242/jcs.109.6.1427

[CIT0039] International Barley Genome Sequencing Consortium. 2012 A physical, genetic and functional sequence assembly of the barley genome. Nature 491, 711–716. 23075845 10.1038/nature11543

[CIT0040] Jackson DA Hassan AB Errington RJ Cook PR 1993 Visualization of focal sites of transcription within human nuclei. EMBO Journal 12, 1059–1065. 8458323 10.1002/j.1460-2075.1993.tb05747.x PMC413307

[CIT0041] Jackson DA Iborra FJ Manders EM Cook PR 1998 Numbers and organization of RNA polymerases, nascent transcripts, and transcription units in HeLa nuclei. Molecular Biology of the Cell 9, 1523–1536. 9614191 10.1091/mbc.9.6.1523 PMC25378

[CIT0042] Jackson DA Pombo A Iborra F 2000 The balance sheet for transcription: an analysis of nuclear RNA metabolism in mammalian cells. FASEB Journal 14, 242–254. 10657981

[CIT0043] Jasencakova Z Meister A Walter J Turner BM Schubert I 2000 Histone H4 acetylation of euchromatin and heterochromatin is cell cycle dependent and correlated with replication rather than with transcription. The Plant Cell 12, 2087–2100. 11090211 10.1105/tpc.12.11.2087 PMC150160

[CIT0044] Kaern M Elston TC Blake WJ Collins JJ 2005 Stochasticity in gene expression: from theories to phenotypes. Nature Reviews Genetics 6, 451–464. 10.1038/nrg1615 15883588

[CIT0045] Kliebenstein DJ West MA van Leeuwen H Kim K Doerge RW Michelmore RW St Clair DA 2006 Genomic survey of gene expression diversity in Arabidopsis thaliana. Genetics 172, 1179–1189. 16204207 10.1534/genetics.105.049353 PMC1456216

[CIT0046] Komarnitsky P Cho EJ Buratowski S 2000 Different phosphorylated forms of RNA polymerase II and associated mRNA processing factors during transcription. Genes and Development 14, 2452–2460. 11018013 10.1101/gad.824700 PMC316976

[CIT0047] Komis G Mistrik M Samajova O Doskocilova A Ovecka M Illes P Bartek J Samaj J 2014 Dynamics and organization of cortical microtubules as revealed by superresolution structured illumination microscopy. Plant Physiology 165, 129–148. 24686112 10.1104/pp.114.238477 PMC4012574

[CIT0048] Kornberg RD 1999 Eukaryotic transcriptional control. Trends in Cell Biology 9, M46–M49. 10611681

[CIT0049] Laird CD Chooi WY 1976 Morphology of transcription units in *Drosophila melanogaster* . Chromosoma 58, 193–218. 826377 10.1007/BF00701359

[CIT0050] Lando D Endesfelder U Berger H 2012 Quantitative single-molecule microscopy reveals that CENP-A(Cnp1) deposition occurs during G2 in fission yeast. Open Biology 2, 120078. 22870388 10.1098/rsob.120078 PMC3411111

[CIT0051] Li GW Xie XS 2011 Central dogma at the single-molecule level in living cells. Nature 475, 308–315. 21776076 10.1038/nature10315 PMC3600414

[CIT0052] Little SC Tikhonov M Gregor T 2013 Precise developmental gene expression arises from globally stochastic transcriptional activity. Cell 154, 789–800. 23953111 10.1016/j.cell.2013.07.025 PMC3778922

[CIT0053] Löschberger A Franke C Krohne G van de Linde S Sauer M 2014 Correlative super-resolution fluorescence and electron microscopy of the nuclear pore complex with molecular resolution. Journal of Cell Science 127, 4351–4355. 25146397 10.1242/jcs.156620

[CIT0054] Markaki Y Gunkel M Schermelleh L Beichmanis S Neumann J Heidemann M Leonhardt H Eick D Cremer C Cremer T 2010 Functional nuclear organization of transcription and DNA replication: a topographical marriage between chromatin domains and the interchromatin compartment. Cold Spring Harb Symposia on Quantitative Biology 75, 475–492. 10.1101/sqb.2010.75.042 21467142

[CIT0055] Martin S Pombo A 2003 Transcription factories: quantitative studies of nanostructures in the mammalian nucleus. Chromosome Research 11, 461–470. 12971722 10.1023/a:1024926710797

[CIT0056] McKnight SL Miller OL Jr 1976 Ultrastructural patterns of RNA synthesis during early embryogenesis of *Drosophila melanogaster* . Cell 8, 305–319. 822943 10.1016/0092-8674(76)90014-3

[CIT0057] Ni Z Schwartz BE Werner J Suarez JR Lis JT 2004 Coordination of transcription, RNA processing, and surveillance by P-TEFb kinase on heat shock genes. Molecular Cell 13, 55–65. 14731394 10.1016/s1097-2765(03)00526-4

[CIT0058] Osborne CS Chakalova L Brown KE 2004 Active genes dynamically colocalize to shared sites of ongoing transcription. Nature Genetics 36, 1065–1071. 15361872 10.1038/ng1423

[CIT0059] Osborne CS Chakalova L Mitchell JA Horton A Wood AL Bolland DJ Corcoran AE Fraser P 2007 Myc dynamically and preferentially relocates to a transcription factory occupied by Igh. PLoS Biology 5, e192. 17622196 10.1371/journal.pbio.0050192 PMC1945077

[CIT0060] Papantonis A Cook PR 2013 Transcription factories: genome organization and gene regulation. Chemical Reviews 113, 8683–8705. 23597155 10.1021/cr300513p

[CIT0061] Pare A Lemons D Kosman D Beaver W Freund Y McGinnis W 2009 Visualization of individual Scr mRNAs during *Drosophila* embryogenesis yields evidence for transcriptional bursting. Current Biology 19, 2037–2042. 19931455 10.1016/j.cub.2009.10.028 PMC2805773

[CIT0062] Park SK Xiang Y Feng X Garrard WT 2014 Pronounced cohabitation of active immunoglobulin genes from three different chromosomes in transcription factories during maximal antibody synthesis. Genes and Development 28, 1159–1164. 24888587 10.1101/gad.237479.114 PMC4052762

[CIT0063] Pennisi E 2012 Genomics. ENCODE project writes eulogy for junk DNA. Science 337, 1159–1161. 22955811 10.1126/science.337.6099.1159

[CIT0064] Pombo A Jackson DA Hollinshead M Wang Z Roeder RG Cook PR 1999 Regional specialization in human nuclei: visualization of discrete sites of transcription by RNA polymerase III. EMBO Journal 18, 2241–2253. 10205177 10.1093/emboj/18.8.2241 PMC1171307

[CIT0065] Raj A Peskin CS Tranchina D Vargas DY Tyagi S 2006 Stochastic mRNA synthesis in mammalian cells. PLoS Biology 4, e309. 17048983 10.1371/journal.pbio.0040309 PMC1563489

[CIT0066] Raj A Rifkin SA Andersen E van Oudenaarden A 2010 Variability in gene expression underlies incomplete penetrance. Nature 463, 913–918. 20164922 10.1038/nature08781 PMC2836165

[CIT0067] Recamier V Izeddin I Bosanac L Dahan M Proux F Darzacq X 2014 Single cell correlation fractal dimension of chromatin: a framework to interpret 3D single molecule super-resolution. Nucleus 5, 75–84. 24637833 10.4161/nucl.28227 PMC4028358

[CIT0068] Reiter M Kirchner B Muller H Holzhauer C Mann W Pfaffl MW 2011 Quantification noise in single cell experiments. Nucleic Acids Research 39, e124. 21745823 10.1093/nar/gkr505 PMC3185419

[CIT0069] Richards CL Rosas U Banta J Bhambhra N Purugganan MD 2012 Genome-wide patterns of Arabidopsis gene expression in nature. PLoS Genetics 8, e1002662. 22532807 10.1371/journal.pgen.1002662 PMC3330097

[CIT0070] Rieder D Trajanoski Z McNally JG 2012 Transcription factories. Frontiers in Genetics 3, 221. 23109938 10.3389/fgene.2012.00221 PMC3478587

[CIT0071] Rossberger S Best G Baddeley D Heintzmann R Birk U Dithmar S Cremer C 2013 Combination of structured illumination and single molecule localization microscopy in one setup. Journal of Optics 15, 094003.

[CIT0072] Schmid M Davison TS Henz SR Pape UJ Demar M Vingron M Scholkopf B Weigel D Lohmann JU 2005 A gene expression map of Arabidopsis thaliana development. Nature Genetics 37, 501–506. 15806101 10.1038/ng1543

[CIT0073] Schubert V 2014 RNA polymerase II forms transcription networks in rye and Arabidopsis nuclei and its amount increases with endopolyploidy. Cytogenetic and Genome Research 143, 69–77. 25060696 10.1159/000365233

[CIT0074] Schubert V Berr A Meister A 2012 Interphase chromatin organisation in Arabidopsis nuclei: constraints versus randomness. Chromosoma 121, 369–387. 22476443 10.1007/s00412-012-0367-8

[CIT0075] Schubert V Lermontova I Schubert I 2013 The Arabidopsis CAP-D proteins are required for correct chromatin organisation, growth and fertility. Chromosoma 122, 517–533. 23929493 10.1007/s00412-013-0424-y

[CIT0076] Schubert V Rudnik R Schubert I 2014 Chromatin associations in Arabidopsis interphase nuclei. Frontiers in Genetics 5, 389. 25431580 10.3389/fgene.2014.00389 PMC4230181

[CIT0077] Sigal A Milo R Cohen A Geva-Zatorsky N Klein Y Liron Y Rosenfeld N Danon T Perzov N Alon U 2006 Variability and memory of protein levels in human cells. Nature 444, 643–646. 17122776 10.1038/nature05316

[CIT0078] Sims RJ 3rd Mandal SS Reinberg D 2004 Recent highlights of RNA-polymerase-II-mediated transcription. Current Opinion in Cell Biology 16, 263–271. 15145350 10.1016/j.ceb.2004.04.004

[CIT0079] Sugden B Keller W 1973 Mammalian deoxyribonucleic acid-dependent ribonucleic acid polymerases. I. Purification and properties of an -amanitin-sensitive ribonucleic acid polymerase and stimulatory factors from HeLa and KB cells. Journal of Biological Chemistry 248, 3777–3788. 4708091

[CIT0080] Taniguchi Y Choi PJ Li GW Chen H Babu M Hearn J Emili A Xie XS 2010 Quantifying *E. coli* proteome and transcriptome with single-molecule sensitivity in single cells. Science 329, 533–538. 20671182 10.1126/science.1188308 PMC2922915

[CIT0081] Tanizawa H Iwasaki O Tanaka A Capizzi JR Wickramasinghe P Lee M Fu Z Noma K 2010 Mapping of long-range associations throughout the fission yeast genome reveals global genome organization linked to transcriptional regulation. Nucleic Acids Research 38, 8164–8177. 21030438 10.1093/nar/gkq955 PMC3001101

[CIT0082] Thompson RE Larson DR Webb WW 2002 Precise nanometer localization analysis for individual fluorescent probes. Biophysical Journal 82, 2775–2783. 11964263 10.1016/S0006-3495(02)75618-X PMC1302065

[CIT0083] Velez-Bermudez IC Schmidt W 2014 The conundrum of discordant protein and mRNA expression. Are plants special? Frontiers in Plant Science 5, 619. 25426129 10.3389/fpls.2014.00619 PMC4224061

[CIT0084] Yan J Enge M Whitington T 2013 Transcription factor binding in human cells occurs in dense clusters formed around cohesin anchor sites. Cell 154, 801–813. 23953112 10.1016/j.cell.2013.07.034

[CIT0085] Zenklusen D Larson DR Singer RH 2008 Single-RNA counting reveals alternative modes of gene expression in yeast. Nature Structural and Molecular Biology 15, 1263–1271. 10.1038/nsmb.1514 PMC3154325 19011635

[CIT0086] Zhang W Zhang T Wu Y Jiang J 2014 Open chromatin in plant genomes. Cytogenetics and Genome Research 143, 18–27. 24923879 10.1159/000362827

[CIT0087] Zhao ZW Roy R Gebhardt JC Suter DM Chapman AR Xie XS 2014 Spatial organization of RNA polymerase II inside a mammalian cell nucleus revealed by reflected light-sheet superresolution microscopy. Proceedings of the National Academy of Sciences, USA 111, 681–686. 10.1073/pnas.1318496111 PMC3896202 24379392

