# Supplementary material for: Abundance and distribution of RNA polymerase II in Arabidopsis interphase nuclei
Source: J Exp Bot. 2015 Mar 4;66(6):1687–98. doi: 10.1093/jxb/erv091 (PMC4357323; doi:10.1093/jxb/erv091)
Supplement: Supplementary Data [file supp_66_6_1687__index.html]

Abundance and distribution of RNA polymerase II in Arabidopsis interphase nuclei — Abundance and distribution of RNA polymerase II in Arabidopsis interphase nuclei — Supplementary Data 

# Abundance and distribution of RNA polymerase II in *Arabidopsis* interphase nuclei

## Supplementary Data

Data files

**Files in this Data Supplement:**

- Supplementary Data - Supplementary Data
