# Supplementary material for: Abundance and distribution of RNA polymerase II in Arabidopsis interphase nuclei
Source: J Exp Bot. 2015 Mar 4;66(6):1687–98. doi: 10.1093/jxb/erv091 (PMC4357323; doi:10.1093/jxb/erv091)
Supplement: Supplementary Data [file supp_erv091_erv091_JEXPBOT_144303_supplementary_data.pdf]

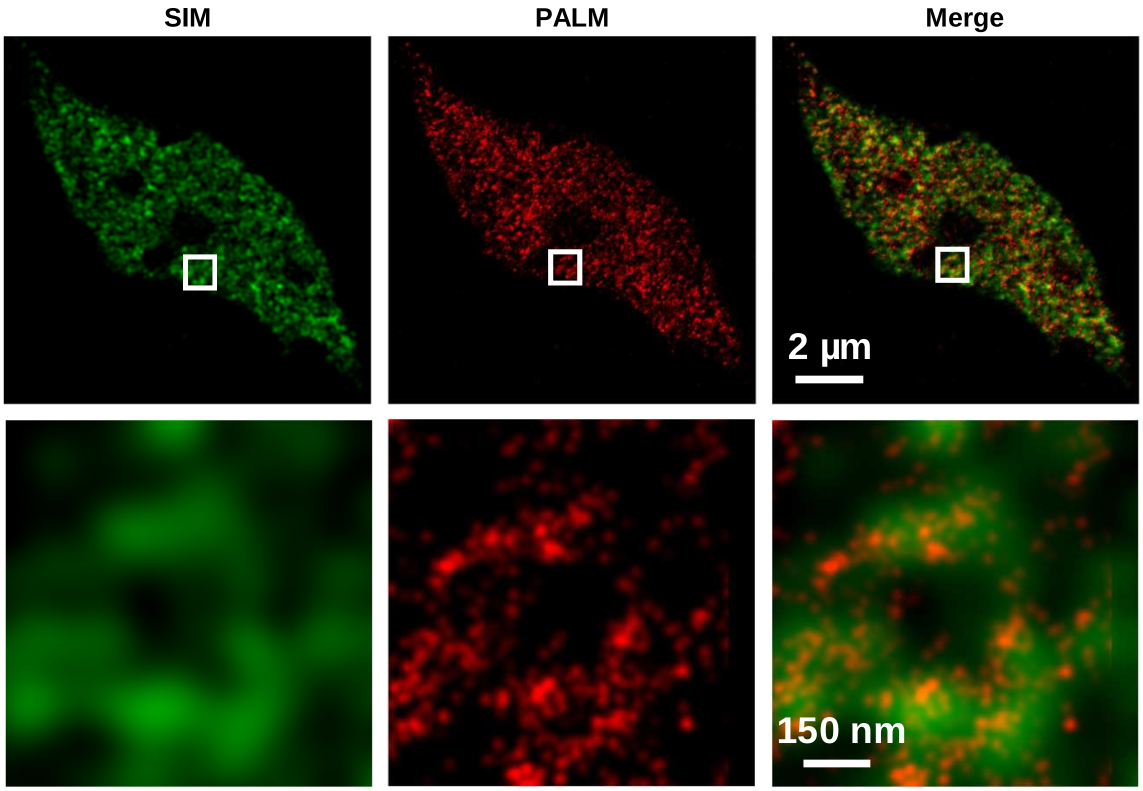

Supplementary Fig. S1. Gauss rendered PALM image of RNAPIIser2ph localized within reticulate structures acquired by SIM. The lower panel shows the boxed area enlarged.

Supplementary Table S1. RNAPII amount in differentiated *A. thaliana* leaf nuclei.
(A) Amount of RNAPIISer2ph in 2C-16C nuclei.
(B) Amount of inactive and active (RNAPIISer2ph, RNAPIISer5ph) RNAPII in 8C nuclei.
(C) Amount of inactiveRNAPII and RNAPIISer2ph in 8C nuclei simultaneously labelled with antibodies against both enzyme modifications.

(A)

	2C	4C	8C	16C
**Mean**	12630	17960	40130	57590
**Std. Dev**	3846	4986	5740	14701
**Min**	6000	10000	29200	32800
**Max**	17960	30500	47300	76000
**No. Nuclei**	11	11	11	11

(B)

	RNAPIIinactive	RNAPIISer2ph	RNAPIISer5ph
**Mean**	22340	54480	52900
**Std. Dev**	10718	14856	10016
**Min**	12500	42300	33000
**Max**	42100	81000	65000
**No. Nuclei**	10	10	10

(C)

	RNAPIIinactive	RNAPIISer2ph	Sum
**Mean**	16640	46690	63330
**Std. Dev**	6006	5528	
**Min**	7400	40100	
**Max**	25150	57000	
**No. Nuclei**	9	9	
**Single Nucleus No.**			
1	15200	53000	68200
2	25150	43200	68350
3	17800	57000	74800
4	23600	46100	69700
5	7400	41600	49000
6	8100	40100	48200
7	16000	46500	62500
8	18700	49200	67900
9	17800	43500	61300
